# Physical-Performance Outcomes and Biomechanical Correlates from the 32-Week Yoga Empowers Seniors Study 

**DOI:** 10.1155/2016/6921689

**Published:** 2016-11-03

**Authors:** Man-Ying Wang, Gail A. Greendale, Sean S.-Y. Yu, George J. Salem

**Affiliations:** ^1^Division of Biokinesiology and Physical Therapy, University of Southern California (USC), 1540 E. Alcazar Street, Los Angeles, CA 90033, USA; ^2^Division of Geriatrics, Geffen School of Medicine, University of California, Los Angeles (UCLA), 924 Westwood Boulevard, Suite 200, Los Angeles, CA 90024, USA

## Abstract

*Background*. Yoga Empowers Seniors Study (YESS) quantified physical demands associated with yoga performance using biomechanical methods. This study evaluated the efficacy of the program on physical function outcomes.* Methods*. Twenty community-dwelling older adults aged 70.7 ± 3.8 years attended biweekly 60-minute Hatha yoga classes for 32 weeks. Four domains of the physical measurements including (1) functional performance, (2) flexibility, (3) muscle strength, and (4) balance were taken at the baseline, 16-week and 32-week time points. Repeated-measures ANOVA omnibus tests and Tukey's post hoc tests were employed to examine the differences in each outcome variable across the 3 time points.* Results*. Improved timed chair stands (*p* < 0.01), 8-foot up and go (*p* < 0.05), 2-min step test (*p* < 0.05), and vertical reach (*p* = 0.05) performance were evident. Isometric knee flexor strength (*p* < 0.05) and repetitions of the heel rise test (*p* < 0.001) also increased following the 32-week intervention. Both flexibility and balance performance remained unchanged.* Conclusions*. Significant improvements in physical function and muscle-specific lower-extremity strength occur with the regular practice of a modified Hatha yoga program designed for seniors. These adaptations corresponded with the previously reported biomechanical demands of the poses.

## 1. Introduction

In the past decade, yoga participation by community-dwelling older adults has continued to rise, increasing from 1.3% in 2002 and 2.0% in 2007 and accelerating to 3.3% in 2012 [1, 2]. Despite its popularity, little is known about the biomechanical demands of yoga participation by seniors or the physical adaptations that occur in association with these demands; thus, the YESS (Yoga Empowers Seniors Study) study was designed to answer these questions. Previous YESS papers have addressed the methodological design of the study and quantified the physical demands of the program poses biomechanically [3–6]; here we report the adaptations in (1) functional performance, (2) flexibility, (3) muscular strength, and (4) balance, following the 32-week modified Hatha yoga program designed specifically for ambulatory older adults.

To date, investigations related to the effects of yoga on physical function, particularly in this cohort, are not conclusive. For example, Chen's group developed the* Silver Yoga* program for seniors and found improved outcomes in functional measures including chair stands, walking speed, and upper- and lower-extremity flexibility, following 24 weeks of yoga participation by older adults (age 69 ± 6.3 yrs) [7]. Similar benefits were also found in assisted-living frail elders and seniors with dementia [8, 9] by the same group of investigators. In addition to improved mobility and flexibility outcomes, beneficial effects of yoga on muscle strength, balance, and fear of falling have also been demonstrated in senior participants in previous studies [10–12]. Unfortunately, these optimistic findings are not consistent across studies. For example, Oken et al. [13] reported that mobility measures including chair stands and timed walking performance did not improve after yoga participation in healthy seniors although enhanced lower-extremity flexibility and balance were evident. Contrastingly, no significant changes in total balance score, fear of falling, flexibility, or quality of life, were found in studies reported by Schmid et al. and Saravanakumar et al. [14, 15].

Several factors such as participant age, physical status, and prior yoga experience and study design including the intervention duration and testing methodology are likely to have contributed to these contradictory findings. The amount of information regarding program design varied greatly across these reports: some only included a list of poses [10, 11, 14]; some provided detailed information of pose progression [15, 16]; some included pose illustrations [12, 17]; others described the programs without pose details [7–9, 13, 18].

Most importantly, none of these prior studies [7–15, 17, 18] quantified the physical demands (joint range of motion, joint moments of force, or muscle activation patterns) of the yoga poses, making it difficult to interpret the mixed results or extrapolate the findings across cohorts. Understanding the links between these physical demands (stimuli) and the participant's physical-performance changes (adaptations) provides a window through which we can begin to appreciate yoga's* mechanisms of action* and allows future yoga instructors and investigators to refine the program in order to maximize its beneficial effects. Put simply, quantifying the stimuli that the body experiences during each pose is one way to decipher why some postures “work” (i.e., in this framework, result in better strength, flexibility, and/or balance) while others do not.

In this study, we adopted a standardized, quantified yoga program, a 32-week yoga intervention program specifically designed for community-dwelling seniors [3]. The aim of this report was to quantify the physical-performance changes, including (1) functional performance, (2) strength, (3) flexibility, and (4) balance, following the yoga intervention. These physical-performance changes are further discussed qualitatively in the context of our reported biomechanical findings measured in the same testing sessions that the final physical function measurements occurred.

## 2. Methods

### 2.1. Study Design

YESS was a single-arm, 32-week, pre-post, intervention-development study. The aims of the study were to quantify both the physical demands of the yoga poses used in the program and the physical-performance adaptations that occurred following the 32-week intervention. The yoga program consisted of 2 phases: a 16-week beginning phase (*Series I*) and a 16-week advanced phase (*Series II*) [3]. The program was designed to be suitable and practical for ambulatory older adults. Anthropometric measurements as well as measures of (1) functional performance, (2) flexibility, (3) muscle strength, and (4) balance were taken at baseline and after each phase of the yoga intervention (a total of 3 measurement sessions: 0 weeks, 16 weeks, and 32 weeks). Data collection was conducted at the Musculoskeletal Biomechanics Research Laboratory (MBRL) at the University of Southern California (USC). Subject recruitment and the yoga classes were conducted at the University of California Los Angeles (UCLA) and TruYoga studio (Santa Monica, CA), respectively. The USC and UCLA Institutional Review Boards approved the study protocol and all participants provided informed, written consent.

### 2.2. Subjects

Community-dwelling older adults, aged 65 years and older, were recruited from the West Los Angeles area via mailing lists, physician referrals, flyers, websites, and newspaper advertisements. Potential subjects were screened via a telephone interview that assessed demographic information, location of residence, transportation capability, and current medical conditions. In order to decrease potential cardiovascular, musculoskeletal, and neurological risks to the participants, related safety exclusions were adopted [3, 4]. Participants also had to execute the following safety tests stably and independently: transition from standing to recumbent on the floor and reverse; lifting both arms to shoulder level; standing with feet side-by-side for 30 seconds; and standing with feet hip-width apart for 60 seconds.

Twenty-four subjects passed the screening exam, were enrolled in the study, and completed the baseline measurements. Twenty of these participants went on to complete the 32-week program and the 2 follow-up assessments (at 16 weeks and 32 weeks). Of the 4 participants who did not complete the intervention, 2 deemed that the time commitment was too great, 1 had recurring posterior thigh pain following the baseline visit (prior to the yoga classes), and one experienced low back pain during the yoga classes (left the study at week 14). The mean percentage of the yoga class attendance over the intervention period was 83% (85.4% ± 7.6% and 80.3% ± 13.2% for* Series I* and* Series II*, resp.). The average age of the 20 participants (6 males and 14 females) was 70.7 ± 3.8 years. Their average height, weight, and body mass index at baseline were 1.67 ± 0.07 m, 71.3 ± 14.6 kg, and 25.3 ± 4.1 kg/m^2^, respectively.

### 2.3. Yoga Program

The program was an adapted form of Hatha yoga that incorporated asanas and* pranayama* (breathing) [19]. It was developed by a research team which included an experienced yoga therapist (EYT-500), a geriatric physician, an exercise physiologist/biomechanist, and a physical therapist. The yoga classes were 60 minutes per session including warm-up and cool-down periods. Classes were held 2 times per week for a total of 32 weeks. Two series of poses,* Series I* and* Series II*, were trained in sequence, each for 16 weeks.

The series were designed to be progressive in nature (i.e., advancing in difficulty) and to train the major muscle groups that are integral to the performance of activities of daily living. The poses for* Series I* included the Chair, Wall Plank, Tree, Warrior I, Warrior II, Downward Facing Dog, Side Stretch, Cobra, Bridge, and Abdominal Cultivation. These classic poses were modified to accommodate the reduced strength, flexibility, and balance capabilities of the senior participants. Modifications included the use of chairs, blocks, and walls, for support. The poses for* Series II* included Chair, Wall Plank, Tree, Warrior II, Side Stretch, Crescent, One-Legged Balance, Recumbent Leg Stretch, Bridge, and Abdominal Cultivation. Poses in* Series II* were performed with fewer modifications, relative to the poses in* Series I*. Additionally, opening (warm-up) poses and finishing (cool-down) poses were incorporated in both series. Detailed pose descriptions and specific modifications, including photos, can be found in a separate report [3]. The report is Open Access and can be viewed via the following links. For the* Series I* poses, see https://www.ncbi.nlm.nih.gov/pmc/articles/PMC3639444/table/T1/. For the* Series II* poses, see https://www.ncbi.nlm.nih.gov/pmc/articles/PMC3639444/table/T2/. Information of the opening and finishing poses are also included in both links. The physical-demand profiles of these poses were detailed in another Open Access paper [4] (https://www.ncbi.nlm.nih.gov/pubmed/24282431).

### 2.4. Measurements

Measurements included tests of (1) functional performance, (2) flexibility, (3) muscle strength, and (4) balance. All measurements were collected at the baseline, at the* Series I* follow-up (16 weeks), and at the* Series II* follow-up (32 weeks).

#### 2.4.1. Functional Performance

Functional performance measurements/tests included (a) timed chair stands, (b) 8-foot up and go, (c) 2-minute step test, (d) horizontal reach, and (e) vertical reach. The timed chair stands test records the number of seconds it takes to stand up 5 times from a chair without using hands [20]. It is an assessment of lower-extremity strength and power and is associated with fall risk and the development of functional dependence [21]. The Intraclass Correlation Coefficient (ICC[2,1]) between weekly measurements for this test in healthy older adults 51–78 years is 0.85. The 8-foot up and go test measures the time it takes for a subject to get up from a chair, walk as quickly as possible around a cone located 8 feet away, return to the chair, and sit down [22]. It assesses agility and dynamic balance and is significantly related to Berg Balance Scale performance, gait speed, and the Barthel Index of ADLs [23]. Performance is also associated with fall risk in older adults [24]. The test-retest reliability for this test in our lab is excellent (ICC[2,1] = 0.95). The 2-minute step test quantifies the number of times that a subject can step in place within 2 minutes [22]. This test measures lower-extremity muscular endurance and is significantly correlated with maximum aerobic capacity, 1-mile walk performance, Balke graded treadmill test, and quality of life [25–28]. The test-retest reliability ICC within our lab is 0.90 in healthy older adults. The maximum horizontal reach test, often referred as functional reach performance, records the maximum forward distance a participant can reach while keeping both feet on the floor [29]. This test evaluates upper- and lower-extremity flexibility, lower-extremity strength, and balance. In order to administer this test, reflective markers were placed on the 3rd metacarpal head. To quantify maximum horizontal reach, the marker position was recorded with an 11-camera motion capture system (Qualisys; Gothenburg, Sweden) while the participant reached forward as far as possible while keeping both feet in contact with the floor. The marker distance from the standing position with the measured arm parallel to the floor to the maximum reach point was calculated and averaged across the 3 trials. This test is significantly correlated with walking speed, social mobility, single-leg standing balance, and fall risk [30, 31]. The maximum vertical reach test which assesses the maximum height a participant can reach during standing was conducted using standard procedures [16]. Similar to the maximum horizontal reach test, the test was measured by tracking the position of the 3rd metacarpal head reflective marker with the motion capture system while the participant reached vertically as high as possible and their feet remained flat on the ground. The highest position of the hand marker was calculated and averaged across 3 attempts. The test-retest reliability for this test is excellent (ICC[2,1] = 0.99) in our laboratory.

#### 2.4.2. Flexibility

Upper- and lower-extremity flexibility were assessed using (a) back scratch and (b) chair sit and reach tests. The back scratch test examines the combined range of motion of the upper-extremity joints in a standing position. Subjects were asked to reach posteriorly with both arms (one superior and other inferior) and attempt to touch or cross their middle fingers across their back [22]. The average distance of overlap (positive value) or distance between the tips of the middle fingers (negative value) across 3 trials was recorded.

The chair sit and reach test assesses upper-extremity (UE), trunk, and lower-extremity (LE, primarily hamstrings) flexibility [22]. Subjects were asked to sit on the edge of a chair with one knee bent at a 90-degree angle (foot flat on the floor), and the other knee extended as straight as possible. Subjects then slowly flexed their trunk and reached forward as far as possible, along their extended limb with overlapped middle fingers. The average distance from the tips of their middle fingers, to the top of their shoe, across 3 trials was recorded.

#### 2.4.3. Muscular Strength and Performance

Muscle strength measures were taken from the following muscle groups of the dominant limb: (a) elbow flexors, (b) elbow extensors, (c) knee flexors, (d) knee extensors, (e) hip abductors, and (f) ankle plantar-flexors. For the elbow and knee muscles, strength was quantified isometrically using the Cybex Norm with HUMAC (CSMi, Stoughton, MA, USA). Standardized testing procedures provided by the manufacturer were employed and standard verbal encouragements were provided. A rest period of 20 seconds was given to the subjects between trials. Subjects practiced 1 warm-up trial and then performed a total of 3 trials for each muscle group. Peak torque during each trial was then recorded and averaged across the trials. Isometric hip abductor strength was measured using the* MicroFET 2 hand held dynamometer* (Hoggan Health Industries, Inc., Draper, UT). Subjects were positioned lying on their side, on an examination table, with their knee and hip extended. A hip strap was placed across the iliac crest to stabilize the pelvis. The dynamometer transducer pad was placed 5 cm proximal to the lateral femoral condyle of the dominant leg (the leg with which they would kick a ball). Subjects were instructed to exert their maximum effort for 5 seconds, 3 times, with 15 second rest intervals between efforts. The peak value during each trial was recorded and averaged across the 3 trials. Ankle plantar-flexor strength and endurance were assessed by quantifying the number of successful heel rise cycles the subject could perform, while standing on the dominant limb, at a speed of 0.5 Hz [32, 33]. Subjects were instructed to rise up onto their toes (plantar-flex their ankle) as many times as possible, to the beat of the metronome (0.5 Hz). The participant was allowed to touch the examiner with a single finger for balance. The test was terminated when the subject (1) failed to lift their heel pass the target mark (1/2 maximum plantar-flexion distance), (2) flexed their knee, (3) requested to stop, or (4) was no longer able to match the movement speed provided by the metronome. Only one trial was administered for this test.

#### 2.4.4. Balance

Balance performance was assessed under the following conditions: (a) double-limb standing with eyes open, (b) double-limb standing with eyes closed, and (c) single-limb standing with eyes open conditions [34–36]. Subjects were requested to stand “quietly” and keep as still as possible, for 2 consecutive 20-second trials in each condition [37]. The tests were ended when the subject moved their feet during double-limb standing tests or touched the ground with their contralateral limb during single-limb standing test. The number of the seconds the subject could perform each task was recorded. If a participant successfully stood for 20 seconds without losing balance, the score was 20 seconds. The average time, across the 2 trials within each condition, was recorded.

### 2.5. Data Analysis

Muscle strength data was normalized to body weight. The differences in each outcome variable among the 3 time points were then examined using repeated-measures ANOVA omnibus tests. When a significant difference was identified, Bonferroni's post hoc tests were used to examine the pairwise comparisons. For all statistically significant post hoc comparisons, Cohen's *d* effect sizes (small* d* = 0.2; medium* d* = 0.5; large* d* = 0.8) are also reported [38]. Statistical analysis was conducted via PASW Statistics 18 (IBM SPSS Statistics, Armonk, NY) and *p* values < 0.05 are considered statistically significance.

## 3. Results

### 3.1. Anthropometrics

Following the 32-week intervention, body weight and body mass index remained unchanged. Body height increased significantly by 0.3% (an average of individual difference = 0.6 cm; *p* < 0.05).

### 3.2. Functional Performance

Results from the repeated-measure ANOVA indicated that timed chair stands (*p* < 0.01), the 8-foot up and go (*p* < 0.05), and the 2-min step test (*p* < 0.05) improved between baseline and follow-up measures (Table 1). A post hoc analysis indicated that the subjects significantly improved timed chair stand performance by 7.8% from the baseline to the 32-week time point (*p* < 0.01,* d* = 0.43). There was an 8.2% improvement in 8-foot up and go performance from week 16 to week 32 (*p* < 0.01,* d* = 0.56). For the 2-min step test, subjects increased by an average of 8.6 repetitions (13.3%) following the 32-week intervention (*p* < 0.05,* d* = −0.50). For the vertical reach, the ANOVA test revealed a borderline significance (*p* = 0.05). When the post hoc analysis was further conducted, results demonstrated that the vertical reach height remained unchanged between baseline and week 16. At the 32-week time point, there was a significant increase (0.3%) in vertical reach height compared to the baseline and the 16-week time points (*p* < 0.001 and *p* < 0.001, resp.). These findings, however, presented with small effect sizes (*d* = −0.11 and −0.11, resp.). There were no significant changes in horizontal reach performance (*p* = 0.46).

### 3.3. Flexibility

There were no significant changes in back scratch test and chair sit and reach test results over time, as indicated by the repeated-measure ANOVA tests (*p* = 0.20, and 0.06, resp., Table 1).

### 3.4. Muscle Strength

**Figure 1:**
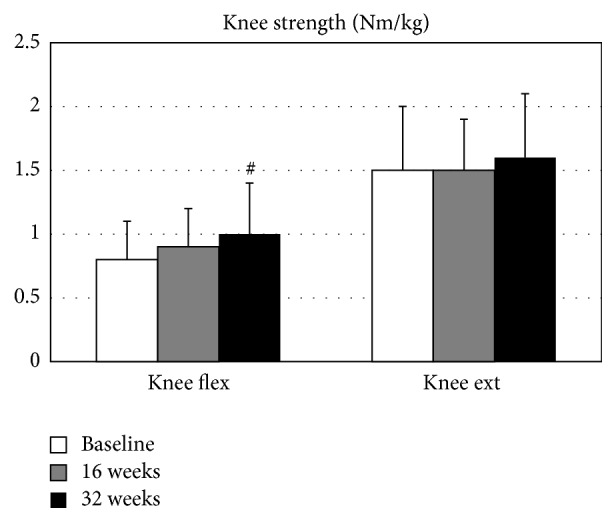
Normalized knee muscle strength.  ^#^Significantly different from the baseline, *p* < 0.05.

**Figure 2:**
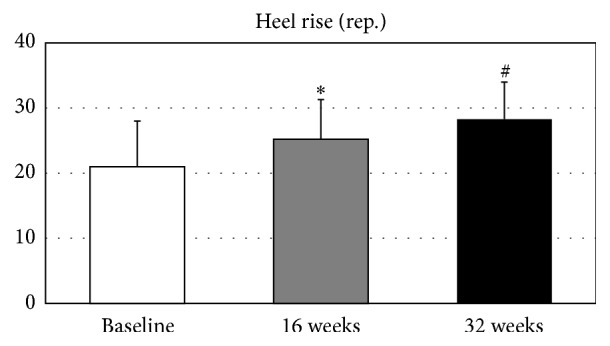
Ankle plantar-flexor strength and endurance.  ^*∗*^Significantly different from the baseline, *p* < 0.05.  ^#^Significantly different from the baseline, *p* < 0.001.

The repeated-measure ANOVA tests demonstrated significant improvement between baseline and follow-up measures for isometric knee flexor strength (*p* < 0.05, Table 2) and the heel rise test (*p* < 0.001). Knee flexor strength increased by 35.8% after 32 weeks of yoga intervention (*p* < 0.05,* d* = −0.57, Figure 1). Likewise, compared to the baseline measures, heel rise performance improved by 29.9% (*p* < 0.05, *d* = −0.64) at week 16 and 45.9% (*p* < 0.001, *d* = −1.14) at week 32 (Figure 2). There were no significant changes found in normalized strength of the elbow flexors (*p* = 0.51), elbow extensors (*p* = 0.13), knee extensors (*p* = 0.42), or hip abductors (*p* = 0.06).

### 3.5. Balance

All subjects were able to stand using both feet (double-limb standing) for the maximum amount of time (20 seconds), with eyes closed and eyes open, at the baseline, 16- and 32-week time points. The average duration of single-limb standing with eyes open at the baseline was 14.5 ± 4.7 seconds. The yoga intervention did not significantly change single-limb standing time (*p* = 0.41).

## 4. Discussion

Hatha yoga is an increasingly popular physical activity adopted by seniors, in part because it is believed to improve and/or preserve physical function. Previous reports on the effects of yoga participation on physical function in seniors have been equivocal and the lack of information regarding the physical demands of these various programs makes it difficult to interpret these conflicting findings. Here we report the physical adaptations, including functional performance, flexibility, muscle strength, and balance, which occurred following a 32-week modified Hatha yoga intervention for seniors. Additionally, we use our previous YESS biomechanical findings, acquired at the same time as the final physical function measurements (32-weeks), to qualitatively interpret our results. The possible clinical implications of the results were summarized in Table 3.

### 4.1. Functional Performance and Flexibility

Functional tests are integrated measures of LE and UE strength, balance, flexibility, speed, power, and reaction time. While each test focuses on specific physical domains (e.g., LE muscular endurance; plantar-flexion function), better performance generally reflects an individual's capacity to accomplish daily living activities, which are paramount to the preservation of independent living [39]. In the current study, the time needed to stand from a chair 5 times dropped by 1 second, a statistically and clinically significant 7.8% improvement, from baseline to the 32-week mark. This finding agrees with results from two recent randomized controlled trials; an 8 wk, 3 d/wk, Hatha yoga study in sedentary adults (age 62.1 ± 5.8 yrs) [10] and a 12 wk, 2 d/wk Iyengar yoga study in community-dwelling seniors (age 67.7 ± 7.2 yrs) [12]. Both studies showed significant improvements in chair stand performance compared to the controls. The chair stands test is significantly correlated with knee strength, walking speed, and lean and fat mass of the LE and UE in independently ambulatory older adults (1,263 women and 1,221 men; aged 70–80 years) [40]. In contrast, slower chair stands time is associated with decreased physical activity level, disability, a history of falls, lower bone mineral density, and fractures [39, 41, 42]. And, not being able to complete this task within 12.9 sec increases the probability of peripheral bone fracture by 2-fold in middle-age women (age 55.1 ± 9.6 yrs, *n* = 484) [39]. Expanding upon this, Khazzani et al. reported that for every 1-second increase in 5 chair stand time, there is a 4% increase in the number of falls per year [39]. Although the rates of falls were not monitored in the current study and our subjects were older compared to Khazzani et al.'s study, the 1.0 sec improvement, from 12.1 sec to 11.1 sec, after the 32-week intervention in the current report, may imply a protective effect against falls. This amount of change approximates to an age difference of 10 years (younger) in normative cross-sectional data by a national survey (*n* = 5,403; age > 60 yrs) [43]. Surprisingly, improvement in timed chair stands performance was not accompanied with changes in quadriceps muscle strength (discussed below), suggesting that other mechanisms (e.g., improved hip extension or ankle plantar-flexion strength, which we observed) may be responsible for this functional improvement.

Participants improved their timed up and go performance by an average of 8.2% between weeks 17–32; however, there was no improvement during the first 16 weeks of yoga practice. These findings suggest that inclusion of the more physically demanding* Series II* poses (which occurred between weeks 17–32) [4–6] is likely necessary to induce improvements in this functional test. The 8-foot up and go test is one of the standard assessments in the Senior Fitness Test developed by Rikli and Jones [22]. In Khazzani et al.'s study, this test, in addition to the timed chair stands test, was a significant predictor of the number of falls per year after adjusting for age [39]. The relative risk was 1.03 falls per one-second increase in timed performance. Moreover, balance, walking speed, and the performance of daily living activities are all correlated with this measure [23, 24]. In the current study, the average individual improvement of the timed up and go test was 0.5 sec. When referencing to the normative cross-sectional data in seniors employing the same standard test, an age difference of 5–10 years (younger) is noted with 0.5 sec difference in performance [22].

In the 2-minute step measure, performance improved by an average of 8.3 repetitions (13.3%) at 32 weeks. This test is a practical field test of aerobic capacity in seniors [28]. Compared to walking test, it requires a greater amount of single-limb support time due to the fact that subjects have to lift their knee above a specified target height (midpoint between the patella and iliac crest). As a result, not only endurance (both muscular and pulmonary) but also balance is assessed. Validations of this step test against cardiopulmonary assessments included maximum aerobic capacity, 1-mile walk test, and Balke graded treadmill test [26, 27]. The measure was also found significantly related to balance performance and risk of falls [44, 45]. The normative cross-sectional data of this 2-min step test were previously reported [22] and with similar amount of improved performance to the current study, an age difference of approximately 15 years (younger) was demonstrated by the cross-sectional data.

A small but statistically significant increase of 0.3% (0.6 cm) in vertical reach distance was observed after 32 weeks. Vertical reach performance is affected by UE and trunk range of motion and strength, balance capabilities, and fear of falling. We did not find changes in the back scratch or sit and reach tests which also test UE flexibility. Thus, we do not believe that increased reach performance occurred because of UE flexibility changes. Unfortunately, the YESS methodology did not include individual measures of trunk flexibility or fear of falling; thus, teasing-out the mechanism underlying the vertical reach improvements is a challenge. While stretching is an important component of yoga pose performance, results are mixed in the literature regarding yoga's effects on flexibility in older adults [7, 8, 15]. Chen and her coworkers developed a 70-min, 3 times per week Silver Yoga program for elders [7, 8, 46]. The summary of their work suggested that improved shoulder range of motion was observed as early as 4 weeks of yoga intervention. The changes in sit and reach performance, however, were not conclusive until 24 weeks of training. On the other hand, after 12 weeks of 75 min, twice weekly, yoga participation, Schmid and colleagues demonstrated no changes in either back scratch or sit and reach performance [15]. Our findings are consistent with Schmid et al.'s report. Factors such as age, initial strength and flexibility, duration of training, program adherence, testing protocols, and asana selections could all affect the results. Our yoga program included many yoga poses that were also incorporated in Schmid et al.'s study, for example, Mountain, Tree, Chair, Warrior I, Warrior II, Side Stretch, and Chair Twist. This comparison was made possible because detailed yoga programs were provided in both studies. Because similar poses were practiced in both studies by similar cohorts, we are not surprised to find comparable results across the two studies. Conversely, details of the Silver Yoga program conducted by Chen's group were not published, making explanations for the result discrepancies difficult to interpret.

### 4.2. Strength and Muscular Performance

Of the 6 muscle performance assessments (elbow flexion/extension, knee flexion/extension, hip abduction, and ankle plantar-flexion), only knee flexion strength and ankle plantar-flexor performance improved after the intervention. Knee flexor strength declines approximately 11% in men and 8% in women, per decade [47]. The 35.8% (0.2 Nm/kg) improvement in knee flexor strength experienced after 32 weeks in the current study was similar to the strength loss that occurs across 2 decades in this age group, according to previous cross-sectional data [47, 48]. Improved knee flexor strength has also been reported to be associated with a reduced incidence of falls in older women [49] and reduced pain and disability in seniors with knee osteoarthritis [50].

Participants also increased their plantar-flexion (heel rise) performance by 45.9% (7.1 repetitions) between the baseline and 32 weeks. With aging, older adults can lose plantar-flexor strength by up to 15% per year after being adjusted for muscle cross-sectional area, physical activity, and gender [51]. Plantar-flexor performance is statistically significantly associated with functional limitations [52], walking speed [53], and risk of falls [54] in community-dwelling seniors. Almost 50% of improvement in plantar-flexion performance in the present study corresponded well with our biomechanics findings collected with the same group of participants at the 32-week point [4, 5], where we reported that* all* of the poses in the yoga program (both* Series I and Series II*) generated internal ankle plantar-flexor joint moments and that none of the poses generated dorsiflexor joint moments. Joint moments are measures of the physical demands of the yoga poses. They are generated by muscular contractions and ligamentous constraints in response to the external moments generated by ground reaction forces. We believe that the high number of plantar-flexor poses we identified in the YESS series resulted in the large training effect identified in the present report.

Our nonsignificant hip abduction-strength changes may also be explained by our previously reported biomechanical findings, the poses that generated significant hip abduction torques, (e.g., single-limb poses like the unsupported Tree pose) were not added until late in the 32-week program [4–6]. Similarly, few of the poses required significant elbow flexor/extensor demands; thus, the nonsignificant changes in these strength measures are also not surprising. The lack of a significant change in knee-extension strength, however, was not expected, given the fact that several of the poses (e.g., Chair, Warrior II, and Crescent) generated relatively high knee extensor demands. These pose demands, however, were not greater than those produced during self-selected walking [5]. Thus, the lack of gains in knee extensor strength may be attributable to an insufficient amount of stimulation (training) provided by the postures in the YESS program.

### 4.3. Balance

Neither standing balance nor horizontal reach performance changed following the yoga intervention. Here again, biomechanical analyses can be used to help us understand these results. Many of the* Series I* poses were modified by allowing subjects to use a wall or chair to assist their balance. When the* Series II* poses were introduced (after week 16) the participants gradually reduced their dependence on the wall and chair, until they could stand on one limb (Tree) or hold a pose without wall support (Side Stretch). Consequently, these more balance-challenging pose versions were used for less than 16 weeks. Likely additional yoga practice beyond 32 weeks and/or the use of more balance-challenging* Series II* poses will be needed to effect changes in these static balance measures.

### 4.4. Limitations and Strengths of the Study

This was not a randomized, controlled trial (RCT); thus we are unable to compare the changes in each of the physical domains to those of an untreated control group. Rather, the study was Phase I,* intervention-development study* which was designed to use biomechanical analyses of the yoga poses, and 16- and 32-week functional performance outcomes from the participants, in order to optimize the design of a future senior yoga program for testing in an RCT. Despite its limited Phase I design, statistically and clinically significant improvements (with large effect sizes) were identified across several important functional tests. The study also was not powered to tease out the effects of sex, diet, body composition, or pose performance skill on these functional performance outcomes; thus, future studies with large samples will be necessary to examine these potential covarying effects.

### 4.5. Summary

This is the first yoga study reporting the physical function adaptations in seniors, when detailed biomechanical profiles of the training poses have been reported. As such, the YESS study and its associated reports are important first steps in unraveling the complicated associations between exercise prescription and physical adaptation in senior yoga science. Our findings suggest that significant improvements in physical function and muscle-specific LE strength occur with the regular practice of a modified Hatha yoga program designed for seniors. Moreover, these adaptations correspond with the biomechanical demands (joint moments and muscle recruitment patterns) of the modified poses. This information can be used to refine the current program by providing a more balanced set of poses, for example, including more dorsiflexor, hip abductor, and single-limb-balance postures, and reducing the number plantar-flexor poses. Future studies, using a RCT design, will be necessary to determine if these biomechanics-based program changes improve outcomes while minimizing adverse events.

## Figures and Tables

**Table 1:** Results for functional performance and flexibility tests (*n* = 20).

Measurement	Baseline (*T*_1_)	16 weeks (*T*_2_)	32 weeks (*T*_3_)	*F*1 (*p*)^a^	Post hoc^b^	
					% change *T*_1_–*T*_3_^c^	% change *T*_2_-*T*_3_^d^
*Functional performance*						
Timed chair Ssand (sec)	12.1 ± 2.3	11.7 ± 2.2	11.1 ± 2.4	5.49 (0.008)	−7.8%^*∗∗*^	−4.6%^ns^
8-foot up and go (sec)	5.2 ± 0.9	5.4 ± 0.9	4.9 ± 0.9	4.79 (0.014)	−5.2%^ns^	−8.2%^*∗∗*^
2 min step (rep.)	75.1 ± 16.7	81.7 ± 17.2	83.7 ± 18.0	3.37 (0.045)	13.3%^*∗*^	3.8%^ns^
Vertical reach (cm)	202.0 ± 10.4	202.0 ± 10.3	203.1 ± 10.5	3.20 (0.052)	0.3%^*∗∗*^	0.3%^*∗∗*^
Horizontal reach (cm)	33.3 ± 5.8	34.0 ± 4.7	34.8 ± 5.2	0.80 (0.455)	—	—
*Flexibility*						
Back scratch (cm)	−5.8 ± 10.1	−5.7 ± 9.4	−4.5 ± 8.9	1.68 (0.201)	—	—
Sit and reach (cm)	−3.9 ± 10.8	−6.1 ± 11.2	−2.5 ± 9.9	2.99 (0.063)	—	—

^a^ *F* and *p* values from repeated-measure ANOVA omnibus tests.

^b^Measurement time points at baseline (*T*_1_), 16 weeks (*T*_2_), and 32 weeks (*T*_3_). No significant differences between *T*_1_ and *T*_2_ were found in all measurements with a significant *F* value. ns = nonsignificant; — = post hoc analysis was not performed because of nonsignificant *F* value.

^c^Percent change was calculated as an average of individual's percent change between *T*_1_ and *T*_3_.

^d^Percent change was calculated as an average of individual's percent change between *T*_2_ and *T*_3_.

^*∗*^ *p* < 0.05.

^*∗∗*^ *p* < 0.01.

**Table 2:** Results for lower-extremity muscle strength tests (*n* = 20).

Strength measure	Baseline (*T*_1_)	16 weeks (*T*_2_)	32 weeks (*T*_3_)	*F*1 (*p*)^a^	Post hoc^b^	
					% change *T*_1_-*T*_2_^c^	% change *T*_1_–*T*_3_^d^
N elbow flex (Nm/kg)	0.6 ± 0.2	0.6 ± 0.2	0.6 ± 0.2	0.70 (0.506)	—	—
N elbow ext (Nm/kg)	0.5 ± 0.2	0.5 ± 0.2	0.5 ± 0.2	2.14 (0.132)	—	—
N knee flex (Nm/kg)	0.8 ± 0.3	0.9 ± 0.3	1.0 ± 0.4	3.61 (0.038)	15.6%^ns^	35.8%^*∗*^
N knee ext (Nm/kg)	1.5 ± 0.5	1.5 ± 0.4	1.6 ± 0.5	0.89 (0.418)	—	—
N hip abd (Nm/kg)	2.6 ± 0.7	2.8 ± 0.5	2.8 ± 0.7	3.14 (0.056)	—	—
Heel rise (rep.)	21.0 ± 7.0	25.2 ± 6.1	28.3 ± 5.7	11.75 (0.000)	29.9%^*∗*^	45.9%^*∗∗*^

N = normalized muscle strength to body weight.

^a^ *F* and *p* values from repeated-measure ANOVA omnibus tests.

^b^Measurement time points at baseline (*T*_1_), 16 weeks (*T*_2_), and 32 weeks (*T*_3_). No significant differences between *T*_2_ and *T*_3_ were found in all measurements with a significant *F* value. ns = nonsignificant; — = post hoc analysis was not performed because of nonsignificant *F* value.

^c^Percent change was calculated as an average of individual's percent change between *T*_1_ and *T*_2_.

^d^Percent change was calculated as an average of individual's percent change between *T*_1_ and *T*_3_.

^*∗*^ *p* < 0.05.

^*∗∗*^ *p* < 0.001.

**Table 3:** Possible clinical implications of the findings in physical adaptations following a 32-week Hatha yoga intervention for seniors.

Measurements	Significant improvement^a^	Possible clinical implication
*Functional performance*		
Timed chair stand	Mild	↑ ADL independence
		↑ gait speed
		↓ disability
		↓ risks of falls
8-foot up and go	Moderate	↑ ADL independence
		↑ balance
		↑ gait speed
		↓ risks of falls
2 min step	Moderate	↑ aerobic capacity
		↑ gait speed
		↑ quality of life
		↓ risks of falls
Vertical reach	Very mild	↑ ADL independence
Horizontal reach	None	No effect
*Strength measures*		
Elbow flexor	None	No effect
Elbow extensor	None	No effect
Knee flexor	Moderate	↓ incidence of falls
		↓ LE disability
Knee extensor	None	No effect
Hip abductor	None	No effect
Heel rise	Major	↑ ADL independence
		↑ gait speed
		↓ risks of falls
*Flexibility*		
Back scratch	None	No effect
Sit and reach	None	No effect
*Balance*		
Single/double limb standing with eyes open/closed	None	No effect

↑ = increase; ↓ = decrease; ADL = activities of daily living; LE = lower-extremity.

^a^Significant improvement after 32 weeks of intervention is categorized by Cohen's *d* effect sizes (mild = *d* > 0.2; moderate = *d* > 0.5; major = *d* > 0.8). None = no statistically significant improvement was found.
